# Supplementary figures and images for: Development of MWCNT/Magnetite Flexible Triboelectric Sensors by Magnetic Patterning
Source: Polymers (Basel). 2023 Jun 29;15(13):2870. doi: 10.3390/polym15132870 (PMC10346752; doi:10.3390/polym15132870)

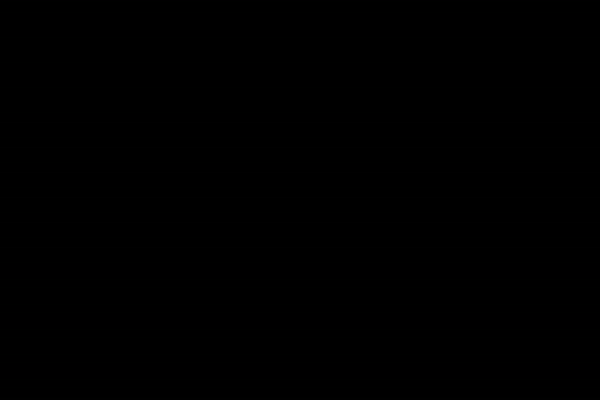

Supplement: Supplementary file 1 [file polymers-15-02870-s001.zip › Fe3O4MWCNT.gif]

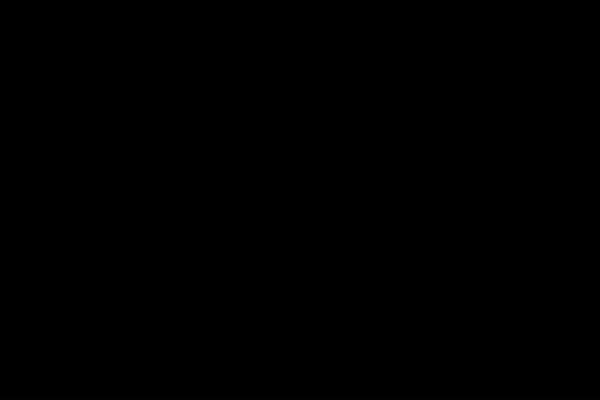

Supplement: Supplementary file 1 [file polymers-15-02870-s001.zip › MWCNT.gif]
